# Lifestyle choices among women with breast cancer in the United States

**DOI:** 10.1002/puh2.153

**Published:** 2024-01-15

**Authors:** Chloé Michel, Michelle Sovinsky, Steven Stern

**Affiliations:** ^1^ Swiss Re Zurich Switzerland; ^2^ Department of Economics University of Mannheim Mannheim Germany; ^3^ Center for Economic and Policy Research Brussels Belgium; ^4^ Department of Economics Stony Brook University Stony Brook New York USA

**Keywords:** breast cancer, health economics, risky health behavior

## Abstract

**Introduction:**

Breast cancer is a common form of cancer for women. The goal of this research was to estimate how a breast cancer diagnosis affects a woman's decisions about smoking, alcohol use, and exercise.

**Methods:**

Using data from the Panel Study of Income Dynamics on breast cancer diagnosis and lifestyle choices, we estimated how being diagnosed influences smoking, drinking, and exercising habits for more than 8000 women over the period 1999–2011.

**Results:**

Controlling for unobserved heterogeneity, persistence in behaviors, and correlation across behaviors, we found that the impact of a diagnosis had a different effect on smoking, drinking, and exercising behaviors. Furthermore, the impact depended upon the recency of the diagnosis. Recently diagnosed women exercised and smoked less—an average woman in our sample reduced exercise by 19% and smoking by 1%. However, women with breast cancer did not change their drinking habits relative to healthy women.

**Conclusions:**

A diagnosis of breast cancer impacts lifestyle choices. Women who were diagnosed with breast cancer in the last 5 years exercised and smoked less but did not change their alcohol consumption after a breast cancer diagnosis regardless of when the diagnosis was made. Our approach provides insight into what extent women who are faced with negative information about life expectancy take this into consideration when deciding to engage in risky behaviors that might further affect their survival. Whether to engage in physical activity, drink alcohol, or smoke are choices associated with how to live.

## Graphical abstract

Controlling for unobserved heterogeneity, persistence in potentially addictive behaviors, and correlation across behaviors, we find robust results that women recently diagnosed with breast cancer exercise and smoke less but do not change their drinking habits relative to healthy women.

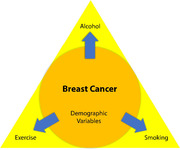

## INTRODUCTION

About 13% of US women will develop breast cancer at some point during their life, and worldwide incidence is rising [1]. There are many genetic and demographic factors linked to breast cancer risk. In addition, several lifestyle habits are associated with incidence, including weight gain, fat intake, and level of physical activity, whereas others have been inconsistently linked with the disease, including alcohol consumption and cigarette smoking [2, 3]. Whether to engage in physical activity, drink alcohol, or smoke are choices associated with how to live [4, 5, 6]. Therefore, understanding lifestyle decisions made by diagnosed women can provide useful information about the tradeoffs women are willing to make between participating in unhealthy habits and increasing one's life expectancy.

Individuals with a breast cancer diagnosis are a particularly informative group to learn about the value of engaging in risky behaviors. Breast cancer is a cancer with one of the highest survival rates—nearly 90% of patients survive the first 5 years. It is also one of the cancers with the highest recurrence rates. Almost 30% of patients with breast cancer who are free of the disease after initial treatment(s) have a recurrence during follow‐up [7]. These facts together suggest that choices made among these individuals can be used to inform us about the value of risky behaviors because (i) behaviors influence incidence, and (ii) there is an incentive to change behavior to combat recurrence.

The Panel Study of Income Dynamics (PSID) contains rich longitudinal information on the timing of breast cancer diagnosis and lifestyle choices among individuals in the United States. We examined the impact a breast cancer diagnosis had on engaging in (potentially addictive) risky behaviors over time. This approach illustrated to what extent women who are faced with negative information about life expectancy take this into consideration when deciding to engage in risky behaviors that might further affect their survival in a significant way.

## METHODS

### Study design and setting

We used data from the PSID, a longitudinal study that started in 1968 and now includes more than 22,000 individuals from over 9000 households in the United States. One person per family, designated as the “head,” is interviewed biennially and answers questions about the individuals of the household. The head of the household provides answers for questions related to his or her spouse. The literature has shown that spouses have very precise perceptions of the time spent by the other spouse on different activities [8]. Similarly, it has been shown that spouses provide complete information for various lifestyle behaviors of their spouse such as smoking and drinking behaviors [9, 10]. Every wave contains information about employment, income, education, wealth, marriage, childbearing, and various other topics. We chose to use the PSID data set because of its longitudinal structure, which allows us to follow the same individuals and their corresponding behaviors across time. Further, these data are collected not only for breast cancer patients but also for persons without a history of cancer. This allows us to make comparisons between breast cancer patients and healthy individuals.

### Sample size and data variables

We used data from seven waves of the PSID from 1999, when cancer outcomes were first recorded, until 2011. We retained respondents who were aged 15 and older and were female because breast cancer almost exclusively affects women. After dropping individuals who had missing information on age, race, education level, income, breast cancer condition, or (lagged) lifestyle behaviors, we had a sample of 8028 women and 34,109 person‐years. Some of these women had missing information on one lifestyle behavior but not another. For our analysis on each behavior, we dropped only those observations with missing values for questions related to those behaviors. So, for smoking habits, this subsample included 8019 women and 33,947 person‐years; for exercise, it included 8009 women and 33,851 person‐years, whereas for drinking, it is smaller (for reasons we discuss momentarily) and included 7175 women and 18,082 person‐years.

### Data handling

In our analysis, we aggregated light and heavy physical activities into a variable called “exercise.” Heavy exercise refers to “heavy housework, aerobics, running, swimming, bicycling or similar activity that causes heavy sweating or large increases in breathing or heart rate” [11]. Light exercise includes “walking, dancing, gardening, golfing, bowling or similar activity that causes only light sweating or slight to moderate increases in breathing or heart rate” [11].

As the survey questions concerning alcohol consumption were not consistently worded across waves, we reported statistics only for the last four waves (2005, 2007, 2009, and 2011). For the first three waves (1999, 2001, and 2003), people were asked how many drinks they had on average per day: “In the last year, on average, how often did you have any alcohol to drink? Would you say, less than one a month, about once a month, several times a month, about once a week, several times a week, or every day?” For the last four waves, the categories were changed, and the questions about daily consumption referred to days when respondents drink: “In the last year, on the days you drank, about how many drinks did you have?” In later regressions, we also used data only from years 2005, 2007, 2009, and 2011 when looking at alcohol behaviors.

With regard to breast cancer diagnosis, the survey asked, “Has a doctor ever told you that you have or had cancer or a malignant tumor?” If the respondent answered “yes,” follow‐up questions were asked regarding the type of cancer and the stage.

### Econometric specifications

In our framework, a woman made a lifestyle choice in each period, where the lifestyle behaviors may have been influenced by breast cancer diagnosis. The lifestyle choices concerned how much to smoke, how much to consume alcohol, and how much to engage in physical activity. We specified equations for each latent variable measuring the continuous quantity of each lifestyle activity chosen by the woman in each time period. Specifically, the baseline model specified each latent dependent variable as a function of lagged behavior, a set of explanatory variables shown in Table 1, whether the woman had breast cancer, a person‐/activity‐specific error, and an idiosyncratic error.

**TABLE 1:** Demographic details of individuals included in the Panel Study of Income Dynamics (1999–2011), United States.

	Total		Breast cancer	
Demographic characteristics^a^	*n*	%	*n*	%
Race				
White	19,838	0.58	560	0.02
Black	10,413	0.31	188	0.01
Married	21,761	0.64	478	–
Employed	21,530	0.63	331	–
Has children	29,668	0.87	730	–
Highest education level				
High school diploma	14,249	0.42	353	–
College degree	11,069	0.33	234	–
Post‐graduate degree	3055	0.09	61	–
Taxable income (US dollars)				
<$20,000	6246	0.18	215	–
$20,001–50,000	20,081		440	–
>$50,001	7782	0.23	146	–
Diagnosed with				
Cancer	3206	0.09	801	1.00
Breast cancer	801	0.02	801	1.00
Current breast cancer status^b^				
Cured	–	–	440	0.75
In remission	–	–	86	0.14
In treatment	–	–	57	0.09

^a^ Number of person‐years = 34,109.

^b^ These questions are asked starting only in 2005; person‐years = 1472; with breast cancer *n* = 583.

There may be heterogeneity that we did not observe in the data that influenced choices and had a persistent nature. Unobserved heterogeneity, likely to influence lifestyle choices, was included as a person‐/behavior‐specific random effect that captured things, such as taste for alcohol or dislike of exercise, and an idiosyncratic effect.

Whether a woman had been diagnosed with breast cancer may have impacted her decision to engage in risky behaviors, for example, if she felt that those behaviors may have reduced her longevity more severely than prior to the breast cancer diagnosis. To the extent that smoking, drinking, or exercise are risk factors for getting breast cancer, one may be concerned that having breast cancer is a function of prior choices. In effect, causation may run in both directions. We addressed issues of endogeneity and unobserved heterogeneity using fixed effects techniques [12]. Finally, we needed to include an initial value of the risky decisions at time *t* = 0. These are likely to be endogenous, and we followed previous literature [12] to control for endogenous initial conditions.

We began by estimating three models corresponding to the lifestyle activities separately. Then we allowed for correlation across smoking, drinking, and exercise behaviors by estimating all decisions jointly. However, due to data restrictions that we mentioned earlier, some of these behaviors are recorded only for a subset of the data. We estimated the parameters of our model by a dynamic ordered probit estimation methodology. Details are provided in Supplementary Files S1 and S2.

## RESULTS

Our sample consisted of 58% white respondents, 30% black respondents, 8% Latino respondents, and 4% from remaining races. The mean age of the respondents was 46.28 (±SD 15.59 years). About 9.4% of the sample had been diagnosed with cancer and 2.3% with breast cancer. The sample average age for a breast cancer diagnosis was approximately 51.39 (±14.73) years. Incidence of breast cancer was not high: We observed 2.3% of the white respondents with breast cancer, 1.8% of the black respondents, and only 0.8% of the Latino respondents. Unfortunately, the sample sizes of Latino respondents and individuals of races other than black and white were too small to allow us to separately identify an effect of being Latino or of another race on behavior. However, individuals of all races are included in our analysis. Our results are interpreted as the impact of being white or black on behavior relative to the impact of being non‐white and non‐black. At the time of the interview, the average year since diagnosis was 11.25 (±11.25) years. Most of our respondents were “cured,” whereas approximately 9% were in treatment. Table 1 reports demographic summary statistics.

The survey reported the proportion of current drinkers, which referred to adults who had at least 12 drinks in their lifetime and at least 1 drink in the past year. Approximately 54% of our respondents ever drank alcoholic beverages for the period 2005–2007, whereas 61% of the white respondents and 43% of the black respondents ever drank alcoholic beverages. Among smokers, breast cancer prevalence was the highest for respondents who smoked more than 19 cigarettes per day. Regarding alcohol consumption behaviors, prevalence was lower in the group of respondents who drank alcohol. Among those who drank, breast cancer prevalence was highest among those women who drank more than one drink per week. The proportion of breast cancer patients was the largest among people who never exercised. Table 2 reports health behavior summary statistics for our sample.

**TABLE 2:** Health behaviors of individuals from the Panel Study of Income Dynamics (1999–2011), United States.

	Total		Breast cancer	
Health behavior	*n*	%	*n*	%
**Smoking status**	33,967			
Current smoker	–	0.18	5987	0.02
**Cigarette consumption**	5987			
Smokes 1–9 cigarettes/day	–	0.33	1998	0.01
Smokes 10–19 cigarettes/day	–	0.36	2174	0.01
Smokes 20 or more cigarettes/day	–	0.30	1815	0.02
**Alcohol**	18,082			
Drinks alcohol	–	0.54	9814	0.02
**Frequency of alcohol consumption** ^a^	9814			
Less than 1 drink/month	–	0.29	2853	0.02
One drink/month	–	0.21	2009	0.02
Several drinks/month	–	0.16	1563	0.02
One drink/week	–	0.17	1640	0.02
Several drinks/week	–	0.14	1346	0.03
Drinks every day	–	0.04	403	0.03
**Exercise**	33,581			
Never	–	0.17	5817	0.04
One or two times/week	–	0.18	6070	0.02
Three–six times/week	–	0.31	10,318	0.02
Seven times/week	–	0.31	10,423	0.02
8–14 times/week	–	0.02	546	0.01
More than 14 times/week	–	0.02	677	0.02

^a^ Used waves 2005–2011.

Table 3 presents random‐effects ordered probit estimates where the explanatory variables included smoking behavior in the previous year, demographics, as well as breast cancer variables. The results show that whether an individual was diagnosed with breast cancer had no significant impact on smoking behavior conditional on past behavior and demographic variables (first two labeled columns). However, as labeled columns (3) and (4) show, if a woman had a diagnosis of breast cancer less than 5 years ago, she would significantly decrease her smoking behavior with this effect being robust to including initial conditions (labeled column 4).

**TABLE 3:** Random‐effects ordered probit regressions for smoking.

Dependent variable: ordered variable for number of cigarettes smoked								
	1		2		3		4	
Variable	Est	*p*	Est	*p*	Est	*p*	Est	*p*
Lagged behavior								
Smoker last period	2.43 (0.03)	<0.01	1.64 (0.04)	<0.01	2.43 (0.03)	<0.01	1.64 (0.04)	<0.01
Breast cancer variables								
Diagnosed with breast cancer	−0.010 (0.09)		−0.15 (0.14)					
Recent breast cancer diagnosis					−0.28 (0.14)	<0.05	−0.32 (0.17)	<0.01
Other controls								
Aged in 30s, 40s, or 50s	−0.02 (0.03)		0.17 (0.05)	<0.05	−0.02 (0.03)		0.17 (0.05)	<0.01
Aged 60s or older	−0.51 (0.05)	<0.01	−0.16 (0.10)		−0.52 (0.05)	<0.01	−0.16 (0.10)	
White	0.47 (0.05)	<0.05	0.70 (0.08)	<0.05	0.47 (0.05)	<0.05	0.70 (0.08)	<0.01
Black	0.08 (0.05)		0.19 (0.08)	<0.05	0.08 (0.05)		0.19 (0.08)	<0.05
Married	−0.22 (0.03)	<0.01	−0.25 (0.03)	<0.01	−0.22 (0.03)	<0.01	−0.25 (0.03)	<0.01
Have children	0.00 (0.04)		−0.03 (0.06)		0.00 (0.04)		−0.03 (0.06)	
Highest education is high school	−0.24 (0.03)	<0.01	−0.35 (0.05)	<0.01	−0.24 (0.03)	<0.01	−0.35 (0.05)	<0.01
Highest education is university degree	−0.50 (0.04)	<0.01	−0.70 (0.06)	<0.01	−0.50 (0.04)	<0.01	−0.70 (0.06)	<0.01
Highest education is post‐graduate	−0.89 (0.07)	<0.01	−1.26 (0.10)	<0.01	−0.89 (0.07)	<0.01	−1.26 (0.10)	<0.01
Income less than $20,000	0.10 (0.03)	<0.01	0.10 (0.04)	<0.01	0.10 (0.03)	<0.01	0.10 (0.04)	<0.01
Income between $20,000 and $50,000	0.09 (0.03)	<0.01	0.10 (0.03)	<0.01	0.09 (0.03)	<0.01	0.10 (0.04)	<0.01
Initial conditions included	No		Yes		No		Yes	
Number of observations	33,967		33,942		33,967		33,942	
Number of individuals	8019		8010		8019		8010	

*Note*: (1) The first columns of each specification give the coefficient estimates with standard errors in parenthesis. (2) The second columns of each specification give the *p*‐values associated with the estimates. (3) All regressions include cutoff points, individual heterogeneity variance, and fixed effects. (4) The initial condition specifications include the mean over time of all time‐varying regressors.

Table 4 presents random‐effects ordered probit estimates for a number of alcoholic drinks, where the dependent variable is ordered according to (i) a nondrinker, (ii) a woman who drinks at most once a week on average, and (iii) a woman who drinks more than once a week on average. As with smoking, we found that past drinking behavior was a positive significant indicator of current drinking behavior, and this effect remained after controlling for initial conditions in labeled columns (2) and (4).

**TABLE 4:** Random‐effects ordered probit regressions for alcohol consumption.

Dependent variable: ordered variable for number of alcoholic drinks								
	1		2		3		4	
Variable	Est	*p*	Est	*p*	Est	*p*	Est	*p*
Lagged behavior								
Number of drinks last period	0.22 (0.01)	<0.01	0.05 (0.01)	<0.01	0.22 (0.01)	<0.01	0.05 (0.01)	<0.01
Breast cancer variables								
Diagnosed with breast cancer	−0.07 (0.13)		−0.10 (0.14)					
Recent breast cancer diagnosis					−0.01 (0.18)		−0.08 (0.18)	
Other controls								
Aged in 30s, 40s, or 50s	0.01 (0.04)		0.10 (0.05)	<0.05	0.01 (0.04)		0.10 (0.05)	<0.01
Aged 60s or older	−0.39 (0.06)	<0.01	−0.16 (0.07)	<0.05	−0.39 (0.06)	<0.01	−0.16 (0.07)	<0.05
White	0.84 (0.0)	<0.01	0.71 (0.07)	<0.01	0.84 (0.07)	<0.01	0.71 (0.07)	<0.01
Black	0.13 (0.07)	<0.10	0.14 (0.08)	<0.10	0.13 (0.07)	<0.10	0.14 (0.08)	<0.10
Married	−0.19 (0.04)	<0.01	−0.15 (0.04)	<0.01	−0.19 (0.04)	<0.01	−0.15 (0.04)	<0.01
Have children	−0.47 (0.05)	<0.01	−0.39 (0.06)	<0.01	−0.47 (0.05)	<0.01	−0.39 (0.06)	<0.01
Highest education is high school	0.46 (0.06)	<0.01	0.46 (0.06)	<0.01	0.46 (0.06)	<0.01	0.46 (0.06)	<0.01
Highest education is university degree	0.80 (0.06)	<0.01	0.80 (0.06)	<0.01	0.80 (0.06)	<0.01	0.80 (0.06)	<0.01
Highest education is post‐graduate	1.00 (0.08)	<0.01	1.01 (0.08)	<0.01	1.00 (0.08)	<0.01	1.01 (0.08)	<0.01
Income less than $20,000	−0.07 (0.04)		−0.05 (0.04)		−0.07 (0.04)		−0.05 (0.04)	
Income between $20,000 and $50,000	−0.06 (0.03)		−0.06 (0.04)		−0.06 (0.03)		−0.06 (0.04)	
Initial conditions included	No		Yes		No		Yes	
Number of observations	18,082		18,036		18,082		18,036	
Number of individuals	7175		7147		7175		7147	

*Note*: (1) The first columns of each specification give the coefficient estimates with standard errors in parenthesis. (2) The second columns of each specification give the *p*‐values associated with the estimates. (3) All regressions include cutoff points, individual heterogeneity variance, and fixed effects. (4) The initial condition specifications include the mean over time of all time‐varying regressors.

We present the results of the random‐effects ordered probit for exercise frequency in Table 5. Exercise frequency was based on the number of exercise sessions per week.

**TABLE 5:** Random‐effects ordered probit regressions for exercising.

Dependent variable: ordered variable for number of exercising								
	1		2		3		4	
Variable	Est	*p*	Est	*p*	Est	*p*	Est	*p*
Lagged behavior								
Exercise frequency last period	0.17 (0.01)	<0.01	0.12 (0.00)	<0.01	0.17 (0.01)	<0.01	0.12 (0.01)	<0.01
Breast cancer variables								
Diagnosed with breast cancer	−0.14 (0.05)	<0.01	−0.16 (0.05)	<0.01				
Recent breast cancer diagnosis					−0.13 (0.07)	<0.05	−0.15 (0.07)	<0.05
Other controls								
Aged in 30s, 40s, or 50s	−0.13 (0.01)	<0.01	−0.14 (0.02)	<0.01	−0.13 (0.01)	<0.01	0.14 (0.02)	<0.01
Aged 60s or older	−0.38 (0.02)	<0.01	−0.39 (0.02)	<0.01	−0.38 (0.02)	<0.01	−0.39 (0.02)	<0.01
White	0.15 (0.02)	<0.01	0.13 (0.02)	<0.01	0.15 (0.02)	<0.01	0.13 (0.02)	<0.01
Black	−0.06 (0.02)	<0.05	−0.06 (0.02)	<0.05	−0.06 (0.02)	<0.05	−0.06 (0.02)	<0.05
Married	0.05 (0.01)	<0.01	0.05 (0.01)	<0.01	0.05 (0.01)	<0.01	0.05 (0.01)	<0.01
Have children	0.01 (0.02)		0.01 (0.02)		0.01 (0.02)		0.01 (0.02)	
Highest education is high school	0.11 (0.02)	<0.01	0.10 (0.02)	<0.01	0.11 (0.02)	<0.01	0.10 (0.02)	<0.01
Highest education is university degree	0.16 (0.02)	<0.01	0.15 (0.02)	<0.01	0.16 (0.02)	<0.01	0.15 (0.02)	<0.01
Highest education is post‐graduate	0.19 (0.03)	<0.01	0.19 (0.03)	<0.01	0.19 (0.03)	<0.01	0.19 (0.03)	<0.01
Income less than 20,000	0.04 (0.02)	<0.05	0.03 (0.02)	<0.10	0.04 (0.02)	<0.05	0.03 (0.02)	<0.10
Income between 20,000 and 50,000	0.06 (0.01)	<0.01	0.05 (0.01)	<0.01	0.06 (0.01)	<0.01	0.05 (0.01)	<0.01
Initial conditions included	No		Yes		No		Yes	
Number of observations	33,851		33,851		33,851		33,851	
Number of individuals	8009		8009		8009		8009	

*Note*: (1) The first columns of each specification give the coefficient estimates with standard errors in parenthesis. (2) The second columns of each specification give the *p*‐values associated with the estimates. (3) All regressions include cutoff points, individual heterogeneity variance, and fixed effects. (4) The initial condition specifications include the mean over time of all time‐varying regressors.

We re‐estimated the specifications from Tables 3, 4, 5 with additional covariates measuring changes. These results are present in Table S1. The results show no significant changes in the impact of a breast cancer diagnosis and the impact of a recent diagnosis on income.

It may be the case that decisions to smoke, drink, or exercise are correlated with each other even after controlling for observed covariates. Table 6 presents the estimates from dynamic multivariate ordered probit regressions that allowed for this correlation. These regressions use information on all behaviors over all periods during which they are available; hence, the sample size is somewhat smaller. The first specification included information on whether an individual was diagnosed with breast cancer, and the second included only a recent diagnosis. We find that there was correlation across behaviors (as evidenced by the significant covariance terms). However, the estimates of the impact of a breast cancer diagnosis remained and were consistent with those from Tables 3, 4, 5.

**TABLE 6:** Multivariate ordered probit regressions.

	1							2					
	Smoking		Drinking			Exercise		Smoking		Drinking		Exercise	
Variable	Est	*p*	Est	*p*		Est	*p*	Est	*p*	Est	*p*	Est	*p*
Lagged behavior	2.53 (0.02)	<0.01	0.43 (0.01)	<0.01		0.29 (0.01)	<0.01	2.53 (0.02)		0.43 (0.01)	<0.01	0.29 (0.00)	<0.01
Diagnosed with breast cancer	−0.06 (0.07)		−0.02 (0.05)			−0.10 (0.03)	<0.01						
Recent breast cancer diagnosis								−0.27 (0.11)	<0.05	0.01 (0.08)		−0.10 (0.06)	<0.10
Aged in 30s, 40s, or 50s	0.02 (0.02)		0.03 (0.02)			−0.12 (0.01)	<0.01	0.03 (0.03)		0.03 (0.03)		−0.12 (0.01)	<0.01
Aged 60 or older	−0.27 (0.03)	<0.01	−0.12 (0.02)	<0.01		−0.32 (0.02)	<0.01	−0.27 (0.03)	<0.01	−0.12 (0.02)	<0.01	−0.33 (0.02)	<0.01
White	0.31 (0.03)	<0.01	0.36 (0.02)	<0.01		0.11 (0.01)	<0.01	0.31 (0.03)	<0.01	0.36 (0.02)	<0.01	0.11 (0.01)	<0.01
Black	−0.03 (0.03)		0.09 (0.02)	<0.01		−0.04 (0.02)	<0.05	−0.03 (0.03)		0.09 (0.02)	<0.01	−0.04 (0.02)	<0.05
Married	−0.13 (0.02)	<0.01	−0.04 (0.01)	<0.05		0.04 (0.01)	<0.01	−0.13 (0.02)	<0.01	−0.04 (0.01)	<0.05	0.04 (0.01)	<0.01
Have children	0.05 (0.03)	<0.10	‐0.17 (0.02)	<0.01		0.01 (0.01)		0.05 (0.03)	<0.10	−0.17 (0.02)	<0.01	0.01 (0.01)	
Highest education is high school	−0.16 (0.02)	<0.01	0.31 (0.02)	<0.01		0.08 (0.01)	<0.01	−0.16 (0.02)	<0.01	0.31 (0.02)	<0.01	0.08 (0.01)	<0.01
Highest education is college degree	−0.36 (0.03)	<0.01	0.57 (0.02)	<0.01		0.10 (0.01)	<0.01	−0.36 (0.03)	<0.01	0.57 (0.02)	<0.01	0.10 (0.01)	<0.01
Highest education is post‐graduate	−0.60 (0.05)	<0.01	0.73 (0.03)	<0.01		0.13 (0.02)	<0.01	−0.60 (0.05)	<0.01	0.73 (0.03)	<0.01	0.13 (0.02)	<0.01
Income less than $20,000	0.07 (0.02)	<0.01	0.07 (0.02)	<0.01		−0.01 (0.01)		0.07 (0.02)	<0.01	0.07 (0.02)	<0.01	−0.01 (0.01)	
Income between $20,000 and $50,000	0.06 (0.02)	<0.05	−0.00 (0.02)			0.00 (0.01)		0.06 (0.02)	<0.05	−0.01 (0.02)		0.01 (0.01)	

Covariance terms	1		2	
Smoking and drinking	0.0478 (0.014)	<0.01	0.0479 (0.014)	<0.01
Smoking and exercise	0.0227 (0.010)	<0.05	0.0227 (0.010)	<0.01
Drinking and exercise	0.0549 (0.009)	<0.01	0.0549 (0.009)	<0.01

*Note*: (1) The first columns of each specification give the coefficient estimates with standard errors in parenthesis. (2) The second columns of each specification give the *p*‐values associated with the estimates. (3) All regressions include cutoff points, individual heterogeneity variance, and fixed effects. (4) The initial condition specifications include the mean over time of all time‐varying regressors.

To put these results in perspective, we evaluated the marginal impact of having a new breast cancer diagnosis on health behaviors for an average woman in our sample. On average, the women in our sample were middle‐aged, white, with a high school education, a household income between $20,000 and $50,000, and married with a child. When the average person in our sample was recently diagnosed with breast cancer, that resulted in an increase in exercising of 19%, a decline in smoking of 1%, and an increase in drinking of 0.2% (but the drinking estimate was not significantly different than zero).

## DISCUSSION

There are numerous studies in the economics and medical literatures that examine issues associated with breast cancer. These include studies on cancer mortality [13], investment in research [14], mammography screening [15], costs of treatment [16], and insurance coverage [8]. However, there are relatively few that consider the relationship with lifestyle choices. Those that do include some focus on smoking [6, 16], some on physical activity [3], and some on alcohol consumption [6]. To the best of our knowledge, ours is the first paper to examine changes in behavior while controlling for persistence in lifestyle choices. Among those papers that examine lifestyle choices among breast cancer survivors, [17] conduct a descriptive analysis of the prevalence of health behaviors (smoking, alcohol use, physical activity, and cancer screening) of cancer survivors by age, time since diagnosis, and cancer site using data from the National Health Interview Survey. They find that cancer survivors are more likely to meet the recommendations for physical activity and cancer screening compared with noncancer controls. (also, see Ref. []). However, they do not find any evidence of different behavior among survivors with respect to smoking and alcohol consumption. We complement and add to previous studies in many ways. First, we use a large, nationally representative sample that includes women diagnosed with breast cancer. Second, we examine changes in lifestyle behaviors over time where we allow for persistence in behavior.

The descriptive statistics showed that breast cancer incidence differed with the degree that an individual engaged in lifestyle behaviors. Our finding that women with recent breast cancer diagnoses significantly reduced their smoking behavior is quite different than the results in Ref. [2]. The differential impact of the time of diagnosis on smoking behavior could arise from a few sources. First, the individual may react to a diagnosis by curbing unhealthy habits such as smoking, but this effect may deteriorate over time as the individual survives past the initial stages. Second, the woman may be undergoing treatment, which makes smoking more difficult in the short term due to lack of energy, for example.

In contrast to smoking behaviors, women did not change their alcohol consumption after a breast cancer diagnosis regardless of when the diagnosis was made, whereas a diagnosis of breast cancer significantly impacted the amount of exercise in a negative way. Perhaps the latter is not so surprising given that women often undergo treatment after a breast cancer diagnosis that can weaken them and make it more difficult to engage in extra physical activity.

## CONCLUSIONS

The impact of diagnosis has a different effect on smoking, drinking, and exercising behavior, and the impact also depends upon the recency of the diagnosis. Women who were diagnosed with breast cancer in the last 5 years smoked less but did not change their alcohol consumption after a breast cancer diagnosis regardless of when the diagnosis was made relative to healthy women. A diagnosis of breast cancer significantly impacted the amount of exercise in a negative way. Perhaps this latter result is not so surprising given that women often undergo treatment after a breast cancer diagnosis that can weaken them and make it more difficult to engage in extra physical activity.

These changes in behavior are not always consistent with information provided to the public on breast cancer risk factors. However, these choices may be rationalized when one considers the overall value of life where lifestyle choices increase the utility from living.

Our approach provided insight into what extent women who are faced with negative information about life expectancy take this into consideration when deciding to engage in risky behaviors that might further affect their survival in a significant way. Whether to engage in physical activity, drink alcohol, or smoke are choices associated with how to live.

## AUTHOR CONTRIBUTIONS

*Conceptualization; data curation; formal analysis; funding acquisition; investigation; methodology; project administration; resources; supervision; validation; visualization; writing—original draft; writing—review and editing*: Michelle Sovinsky and Steven Stern.

## CONFLICT OF INTEREST STATEMENT

We have no conflicts of interest to disclose.

## ETHICS STATEMENT

None

## Supporting information

Supporting Information

Supporting Information

Supporting Information

Supporting Information

## Data Availability

Panel Study of Income Dynamics data is publicly available, for example, through the Inter‐university Consortium for Political and Social Research (ICPSR).
