# Supplementary material for: Lifestyle choices among women with breast cancer in the United States
Source: Public Health Chall. 2024 Jan 15;3(1):e153. doi: 10.1002/puh2.153 (PMC12060761; doi:10.1002/puh2.153)
Supplement: Supplementary file 1 — Supporting Information [file PUH2-3-e153-s003.docx]

**DETAILS OF ECONOMETRIC SPECIFICATION**

In our framework, a woman (indexed by i) makes a lifestyle choice (indexed by l) in each period (indexed by t), where the lifestyle behaviors may be influenced by breast cancer diagnosis. The lifestyle choices concern how much to smoke, how much to consume alcohol, and how much to engage in physical activity. Let $y_{ilt}^{*}$ be a latent variable measuring the continuous quantity of lifestyle activity l chosen by individual i at time t. Specifically, the baseline model is given by

$y_{ilt}^{*}=1\left( y_{ilt-1}>0 \right)\alpha_{l}+X_{it}\eta_{l}+b_{it}\delta_{l}+\mu_{il}+\varepsilon_{ilt} (1)$

where $1\left( y_{ilt-1}>0 \right)$ is an indicator function which is equal to 1 if the condition inside the parentheses is true and 0 otherwise. Lifestyle choices exhibit persistence, which may be due to addiction (such as smoking and drinking alcohol) or habit persistence (such as exercise). Therefore, we allow individual i's lifestyle choices at time t to depend on whether she participated in that behavior in the immediate past $1\left( y_{ilt-1}>0 \right)$. Exogenous, possibly time-varying individual demographic variables $X_{it}$ include i's age, her marital status, whether she has children, her income, and her education level.

There may be heterogeneity that we do not observe in the data that influences choices and has a persistent nature. Unobserved heterogeneity likely to influence lifestyle choices includes a person/behavior-specific random effect $\mu_{il}$ which captures things such as taste for alcohol or dislike of exercise and an idiosyncratic effect $\varepsilon_{ilt}$.

Whether a woman has been diagnosed with breast cancer (captured by dummy $b_{it}$) may impact her decision to engage in risky behaviors. To the extent that smoking, drinking, or exercise are risk factors for getting breast cancer, one may be concerned that $b_{it}$ is a function of prior choices. In effect, causation may run in both directions. We address issues of endogeneity and unobserved heterogeneity using fixed effects techniques [13]. The remaining objects in equation (1) are parameters to estimate.

Finally, we need to include an initial value of the risky decisions at time t=0. These are likely to be endogenous, and we follow previous literature [19] to control for endogenous initial conditions. We specify the initial period values as

$y_{il0}^{*}=C_{i}\varsigma_{l}+X_{i0}\eta_{l}+b_{i0}\delta_{l}+\mu_{il}+\varepsilon_{il0}$ (2)

$\varepsilon_{il0}\sim iid$N(0,$\sigma_{\varepsilon}^{2}),$

where $C_{i}$ is a set of variables affecting only initial choices and $\varsigma_{l}, \eta_{l},$ $\sigma_{\varepsilon}^{2},$and $\delta_{l}$ are parameters to be estimated. For smoking behaviors, these include when i started smoking. For those who do not smoke, the initial condition is set to zero. This is an innocuous normalization because we control for those who have never smoked. A separate concern is that the age when one started smoking might be endogenous. However, most of the literature [20] ignores this issue. Unfortunately, the PSID does not contain any information on the age at which respondents started drinking or exercising. For these lifestyle choices, we include the level of drinking or exercising behavior observed in the first period of the data as $C_{i}$. In this approach, there may be a concern about the value of $\mu_{il}$. One possibility is to treat it as random, which would imply that $\mu_{il}$ and $y_{il0}^{*}$ are independent. However, $\mu_{il}$ and $y_{il0}^{*}$ may not be independent, so we specify the construction of the fixed effect conditional on the initial condition as

$\mu_{il}=\pi_{0}+\pi_{1}y_{il0}^{*}+X_{i}\pi_{2}+v_{il}$ (3)

$v_{il}\sim iidN(0, \sigma_{v}^{2})$

where $X_{i}$ denotes the mean over time of the explanatory variables (excluding the year fixed effects) [13, 21]. The random component of the fixed effect then can be integrated out to yield the likelihood function of the random effects probit model with time t, observation i explanatory variables: ($X_{it}$, $y_{ilt-1}{,..,y}_{il0}$, $X_{i}$) [13] (we define $y_{ilt}$ momentarily).
