# Supplementary material for: Lifestyle choices among women with breast cancer in the United States
Source: Public Health Chall. 2024 Jan 15;3(1):e153. doi: 10.1002/puh2.153 (PMC12060761; doi:10.1002/puh2.153)
Supplement: Supplementary file 2 — Supporting Information [file PUH2-3-e153-s004.docx]

**DETAILS OF ECONOMETRIC METHODOLOGY**

We estimate three models corresponding to the lifestyle activities separately. Then, we allow for correlation across smoking, drinking, and exercise behaviors by estimating all decisions jointly. We estimate the parameters of our model by a straightforward dynamic ordered probit estimation methodology.

In the context of our framework, using equation (1), we define

$$Y_{ilt}\left( \mu_{il} \right)=1\left( y_{ilt-1}^{*}>0 \right)\alpha_{l}+X_{it}\eta_{l}+b_{it}\delta_{l}+\mu_{il}$$

as the deterministic part of $y_{ilt}^{*}$ after conditioning on $\mu_{il}$ for t≥1. Then, using equation (2), we can define

$$Y_{il0}\left( \mu_{il} \right)=C_{i}\varsigma_{l}+X_{i0}\eta_{l}+b_{i0}\delta_{l}+\mu_{il}$$

where, using equation (3), we define

$$\mu_{il}=\pi_{0}+\pi_{1}y_{il0}^{*}+X_{i}\pi_{2}.$$

There is only one small complication: each behavior is reported in the data as a bracketed variable. To account for this, we define m=1 as not participating in the activity and let the quantity of the activity increase as m increases with

$y_{ilt}=m iff \kappa_{lm}\leq y_{ilt}^{*}<\kappa_{lm+1}, m=1,2,..,M_{l}$ (4)

where $\kappa_{lm}$ are cutoff points to be estimated and, without loss of generality, that $\kappa_{l1}$=-∞, $\kappa_{l2}$=0, and $\kappa_{M_{l}+1}$=∞.; this is akin to a dynamic ordered probit model. The vector of parameters to estimate for model l is $\theta_{l}=(\alpha_{l},\eta_{l},\varsigma_{l},\pi_{0},$ $\pi_{1},$ $\pi_{2},$ $\sigma_{\varepsilon},\sigma_{v},\kappa_{l})$, the conditional likelihood contribution for lifestyle choice l individual i is

$$L_{il}\left( \mu\right)=\prod_{t=1}^{T} \left( \prod_{m=1}^{M_{l}} \Delta_{ilm}(\mu)^{1\left( y_{ilt}=m \right)} \right)\left( \prod_{m=1}^{M_{l}} \Delta_{ilm}(\frac{\mu}{\sigma_{\varepsilon}})^{1\left( y_{il0}=m \right)} \right)\frac{1}{\sigma_{v}}\phi\left( \frac{\mu-\bar{\mu}_{il}}{\sigma_{v}} \right)$$

where

$$\Delta_{ilm}\left( \mu\right)=\Phi\left( \frac{\kappa_{lm+1}-Y_{ilt}(\mu)}{\sigma} \right)-\Phi\left( \frac{\kappa_{lm}-Y_{ilt}(\mu)}{\sigma} \right)$$

for σ=1 or $\sigma=\sigma_{\varepsilon}$, and the log likelihood contribution is

$${logL}_{il}=log\int L_{il}\left( \mu\right)d\mu.$$

The log likelihood function $logL_{l}=\sum_{i} logL_{il}$ can be evaluated using a quadrature method [22]. Also, we use antithetic acceleration in simulation. The loss of precision in simulation is of the order 1/N (where N is the number of observations) when antithetic acceleration is used [23], which does not require an adjustment to the asymptotic covariance matrix.
