# Supplementary material for: Lifestyle choices among women with breast cancer in the United States
Source: Public Health Chall. 2024 Jan 15;3(1):e153. doi: 10.1002/puh2.153 (PMC12060761; doi:10.1002/puh2.153)
Supplement: Supplementary file 3 — Supporting Information [file PUH2-3-e153-s001.docx]

**MULTIVARIATE ORDERED PROBIT**

To allow for correlation across risky lifestyle choices, we estimate a (dynamic) multivariate ordered probit model. To allow for correlation across choices in the error structure, we estimate a dynamic multivariate ordered probit model where

$$\varepsilon_{it}=\left( \begin{matrix} \varepsilon_{i1t} \\ \varepsilon_{i2t} \\ \varepsilon_{i3t} \end{matrix} \right)\sim iidN\left[ 0,\Omega\right]$$

with

$$\Omega=\left( \begin{matrix} 1 & \rho_{12} & \rho_{13} \\ \rho_{12} & 1 & \rho_{23} \\ \rho_{13} & \rho_{23} & 1 \end{matrix} \right),$$

the vector of parameters is augmented to include (ρ₁₂, ρ₁₃, ρ₂₃), the conditional likelihood contribution for individual i is

$$L_{i}\left( \mu_{i} \right)=\prod_{t=1}^{T} \left( \prod_{\vec{m}} {\Delta_{i\vec{m}}\left( \mu_{i} \right)}^{1\left( y_{it}=\vec{m} \right)} \right)\left( \prod_{\vec{m}} {\Delta_{i\vec{m}}\left( \mu_{i} \right)}^{1\left( y_{i0}=\vec{m} \right)} \right),$$

where $\vec{m}$=(m₁, m₂, m₃) is the vector of discrete choices made for each risky behavior, $\mu_{i}=\left( \mu_{i1}, \mu_{i2},\mu_{i3} \right),$ $y_{it}=\left( y_{i1t,}y_{i2t,}y_{i3t,} \right)$, and

$$\Delta_{\vec{m}}\left( \mu_{i} \right)=Pr\left[ \kappa_{lm_{l}}-Y_{ilt}\left( \mu_{il} \right)\leq\varepsilon_{ilt}<\kappa_{lm_{l+1}}-Y_{ilt}\left( \mu_{il} \right), l=1,2,3 \right]$$

which is the trivariate normal density with covariance matrix Ω integrated over the rectangle with sides defined by the limits of $\varepsilon_{ilt}$. The log likelihood contribution for i is

$$logL_{i}=log\int L_{i}\left( \mu_{i} \right)\prod_{l=1}^{3} \frac{1}{\sigma_{v}}\phi\left( \frac{\mu_{il}}{\sigma_{v}} \right)d\mu_{il}.$$
