# Supplementary material for: Lifestyle choices among women with breast cancer in the United States
Source: Public Health Chall. 2024 Jan 15;3(1):e153. doi: 10.1002/puh2.153 (PMC12060761; doi:10.1002/puh2.153)
Supplement: Supplementary file 4 — Supporting Information [file PUH2-3-e153-s002.docx]

**ESTIMATES FOR OTHER SPECIFICATIONS**

| Supplementary Table 1: Regressions Including Changes in Covariates | | | | | | | | | | | | |
| --- | --- | --- | --- | --- | --- | --- | --- | --- | --- | --- | --- | --- |
|  | | | | | | | | | | | | |
|  | Risky Behavior | | | | | | | | | | | |
|  | Smoking | | Smoking | | Drinking | | Drinking | | Exercise | | Exercise | |
| Lagged Behavior | 2.428 | *** | 2.430 | *** | 0.219 | *** | 0.219 | *** | 0.174 | *** | 0.174 | *** |
|  | (0.030) |  | (0.030) |  | (0.011) |  | (0.011) |  | (0.007) |  | (0.007) |  |
| Diagnosed with Breast Cancer | -0.097 |  |  |  | -0.068 | *** | |  | -0.130 | *** | |  |
|  | (0.073) |  |  |  | (0.039) |  |  |  | (0.050) |  |  |  |
| Recent Breast Cancer Diagnosis |  |  | -0.288 | *** | |  | 0.002 |  |  |  | -0.128 | * |
|  |  |  | (0.140) |  |  |  | (0.180) |  |  |  | (0.070) |  |
| Change in Marital Status | 0.150 | *** | 0.149 | *** | -0.006 |  | -0.006 |  | 0.023 |  | 0.023 |  |
|  | (0.039) |  | (0.039) |  | (0.051) |  | (0.051) |  | (0.024) |  | (0.024) |  |
| Moved into Poor Health | 0.138 | ** | 0.137 | ** | -0.363 | *** | -0.364 | *** | -0.548 | *** | -0.549 | *** |
|  | (0.068) |  | (0.068) |  | (0.094) |  | (0.094) |  | (0.043) |  | (0.043) |  |
| Change in Employment Status | 0.066 | ** | 0.066 | ** | -0.053 |  | -0.053 |  | 0.050 | *** | 0.050 | *** |
|  | (0.029) |  | (0.029) |  | (0.037) |  | (0.037) |  | (0.017) |  | (0.017) |  |
| Aged in 30s, 40s, or 50s | -0.013 |  | -0.012 |  | 0.017 |  | 0.017 |  | -0.124 | *** | -0.124 | *** |
|  | (0.033) |  | (0.033) |  | (0.048) |  | (0.048) |  | (0.020) |  | (0.020) |  |
| Aged 60 or Older | -0.494 | *** | -0.495 | *** | -0.379 | *** | -0.383 | *** | -0.349 | *** | -0.354 | *** |
|  | (0.016) |  | (0.016) |  | (0.068) |  | (0.067) |  | (0.026) |  | (0.026) |  |
| White | 0.480 | *** | 0.480 | *** | 0.848 | *** | 0.847 | *** | 0.151 | *** | 0.150 | *** |
|  | (0.053) |  | (0.053) |  | (0.074) |  | (0.074) |  | (0.026) |  | (0.026) |  |
| Black | 0.090 |  | 0.089 |  | 0.133 | * | 0.133 | * | -0.067 | ** | -0.068 | ** |
|  | (0.055) |  | (0.055) |  | (0.078) |  | (0.078) |  | (0.027) |  | (0.027) |  |
|  |  |  |  |  |  |  |  |  |  |  |  |  |

|  |  |  |  |  |  |  |  |  |  |  |
| --- | --- | --- | --- | --- | --- | --- | --- | --- | --- | --- |

| Supplementary Table 1 (Continued) | | | | | | |
| --- | --- | --- | --- | --- | --- | --- |
|  | Risky Behavior | | | | | |
|  | Smoking | Smoking | Drinking | Drinking | Exercise | Exercise |

| Married | -0.219 | *** | -0.219 | *** | -0.196 | *** | -0.196 | *** | 0.051 | *** | 0.051 | *** |
| --- | --- | --- | --- | --- | --- | --- | --- | --- | --- | --- | --- | --- |
|  | (0.030) |  | (0.030) |  | (0.043) |  | (0.043) |  | (0.017) |  | (0.017) |  |
| Have Children | -0.004 |  | -0.004 |  | -0.475 | *** | -0.476 | *** | 0.015 |  | 0.014 |  |
|  | (0.042) |  | (0.042) |  | (0.059) |  | (0.059) |  | (0.023) |  | (0.023) |  |
| Highest Education is High School | -0.237 | *** | -0.237 | *** | 0.458 | *** | 0.458 | *** | 0.099 | *** | 0.099 | *** |
|  | (0.036) |  | (0.036) |  | (0.061) |  | (0.061) |  | (0.022) |  | (0.022) |  |
| Highest Education is College Degree | -0.504 | *** | -0.504 | *** | 0.795 | *** | 0.795 | *** | 0.147 | *** | 0.147 | *** |
|  | (0.042) |  | (0.042) |  | (0.064) |  | (0.064) |  | (0.024) |  | (0.024) |  |
| Highest Education is Post Graduate | -0.896 | *** | -0.895 | *** | 0.997 | *** | 0.998 | *** | 0.178 | *** | 0.178 | *** |
|  | (0.075) |  | (0.075) |  | (0.082) |  | (0.082) |  | (0.032) |  | (0.032) |  |
| Income Less than 20K | 0.087 | ** | 0.087 | ** | -0.076 | * | -0.076 | * | 0.043 | ** | 0.043 | ** |
|  | (0.035) |  | (0.035) |  | (0.046) |  | (0.046) |  | (0.020) |  | (0.020) |  |
| Income Between 20K and 50K | 0.090 | *** | 0.089 | *** | -0.066 | * | -0.065 | * | 0.059 | *** | 0.059 | *** |
|  | (0.031) |  | (0.031) |  | (0.039) |  | (0.039) |  | (0.017) |  | (0.017) |  |
| Number of Observations | 33,967 |  | 33,967 |  | 18,082 |  | 18,082 |  | 33,851 |  | 33,851 |  |
| Number of Individuals | 8,019 |  | 8,019 |  | 7,175 |  | 7,175 |  | 8,009 |  | 8,009 |  |
| Notes: |  |  |  |  |  |  |  |  |  |  |  |  |
| 1) Standard errors are in parentheses. | |  |  |  |  |  |  |  |  |  |  |  |
| 2) * p-value<.10, ** p-value<.05, *** p-value<.01 | | |  |  |  |  |  |  |  |  |  |  |
| 3) All regressions include cutoff points, individual heterogeneity variance, and fixed effects. | | | | | | | | |  |  |  |  |
